# Volatile Organic Compounds of Anchote Tuber and Leaf Extracted Using Simultaneous Steam Distillation and Solvent Extraction

**DOI:** 10.1155/2022/3265488

**Published:** 2022-09-13

**Authors:** Yenenesh Ayalew, Dargie Tsegay Berhe, Nigusse Retta, Gulelat Desse, Ali Mohammed, Kyong Su Kim

**Affiliations:** ^1^Dilla University, College of Agriculture & Natural Resources, Dilla, Ethiopia; ^2^Addis Ababa University, College of Natural Sciences, Addis Ababa, Ethiopia; ^3^Botswana University, Gaborone, Botswana; ^4^Jimma University, College of Agriculture and Veterinary Medicine, Jimma, Ethiopia; ^5^Chosun University, Department of Food and Nutrition, Gwangju 501-759, Republic of Korea

## Abstract

Anchote (*Coccinia abyssinica* (Lam.) (Cogn)) is an endemic and potentially valuable crop of Ethiopia principally categorized under root and tuber crops, and its newly growing leaves along with the tendrils are also used as nutritious vegetable served after being cooked. Leaf and tuber powders were extracted for the first time to identify volatile organic compounds by simultaneous steam distillation and solvent extraction (SDE) and characterized using gas chromatography mass spectrometry (GC-MS). VOCs having an area percentage above 0.5% were used for identification analysis. From the results, thirty volatile flavor compounds from leaves and fifteen from tubers were identified with the total fraction yield of 770.57 mg/kg and 4536.91 mg/kg, respectively, and from the 30 compounds identified from leaf 16 were distinguished in each of the tested accessions. Ethyl acetate 90.47% (697.13 mg/kg) was detected in a higher amount exhibiting >1% peak area. The rest 6.03% (46.46 mg/kg) were minor quantities (<1%) of the total (770.57 mg/kg) volatile flavor fraction. Among the 15 identified compounds in the tuber, ethyl acetate was the only major compound that accounted together for 99.15% (4498.33 mg/kg) of the total volatile flavor fraction and 0.85% (38.58 mg/kg) being reported in minor quantities (<1%). The SDE extraction and GC-MS analysis of anchote leaves and tubers successfully identified various volatile flavor compounds, which indicates that anchote was found to be a potential source of volatile flavor compounds that can be used as a food flavoring agent and in folk medicines. Thus, this study confirms that anchote leaf and tuber can be used for more specific and valuable applications in food and medicine industries.

## 1. Introduction

More than 1,000 low molecular weight (*M*_*r*_) organic compounds are emitted from floral and vegetative parts of many plant species [1]. The release of volatiles from many vegetative organs has antimicrobial or antiherbivore activity and so could also act to protect valuable reproductive parts of plants from enemies [2, 3].

Volatile organic compounds (VOCs) have great importance in basic and applied research. Plant parts release VOCs into the atmosphere and the soil as a defense mechanism against herbivores and pathogens or as an attraction of pollinators and seed dispersers. In some plants, released VOCs may also act as wound sealers [4].

Flavor, taste, and sensorial quality of food, in which the aroma is formed by a complex group of chemical substances, are influenced by VOCs. Over 90% of the natural emission of VOCs depends on plant species, and aroma results from the interplay of these emitted VOCs [4, 5]. Identification of VOCs for fragrance, pharmacologically active ingredients, and for flavor compounds is important for potential multipurpose functional use [6, 7]. VOCs formed biochemically during ripening of root bulbs, stem bark, fruits, and other vegetables [8], and among them, only a limited number are important for the characteristic aroma of the food, which are called key odorants [9].

The widely used simultaneous distillation extraction (SDE) technique is the most traditional extraction method, versatile, and relatively simple; however, the high temperature applied during the process, as well as long extraction time, might lead to artifact formation. In certain cases, SDE can provide bigger peaks of thermally stable compounds. SDE is widely applied for food products [10].

Anchote (*Coccinia abyssinica* (Lam.) (Cogn.)) is one of the important endemic crops principally grown for its edible tubers throughout the south and southwestern parts of Ethiopia. Moreover, its newly grown leaves along with tendrils are served as green vegetable after cooking. It has been used as a traditional medicine for a long period of time to treat various illnesses [11, 12]. Traditional use of anchote is for a number of medicinal reasons including fast mending of broken bones due to its good content of calcium [10] and for making lactating mothers healthier and stronger as well as to treat gonorrhea, tuberculosis, and tumor cancer [13]. Knowing the volatile flavor of anchote would be essential in discovering the key compounds responsible for the aroma and health effects. Therefore, the objective of the present study was to determine the active components and their contribution towards the overall aroma impact and medicinal values of anchote leaves and tubers.

## 2. Materials and Methods

### 2.1. Reagents

All reagents used in this experiment were purchased from Sigma Co. (St. Louis, USA) and Fisher Scientific (Waltham, Massachusetts, USA). The organic solvents: diethyl ether and n-pentane were redistilled using a wire spiral packed double distilling apparatus (Normschliff Geratebau, Wertheim, Germany). Besides, Milli-Q water was generated through a water purification system (Millipore Corporation, Bedford, USA), and the anhydrous Na_2_SO_4_ was used for dehydration of organic solvents after burning overnight at 650°C in a furnace (F 6000, Barnstead Thermolyne Co., IA, USA) and allowed to cool down in desiccators.

### 2.2. Extraction of Volatile Organic Compounds

Three *anchote* accessions, “223090,” “220563,” and “230566,” were selected for volatile flavor profile studies. Three healthy tubers from each accession were harvested from Debre Zeit Agricultural Research Center (DZARC) experimental field in June 2014, washed, peeled, and sliced to small pieces and mixed thoroughly in order to prepare 400 g of samples. Similarly, 200 g bunches of newly growing tips of leaves were first cleaned and chopped into small pieces. Both tuber and leaf samples were oven dried at 105°C to a constant weight in a paper bag by a hot air oven (DHG-9055A, Memment, Germany) set at 105°C. The oven dried anchote leaf and tuber samples were then milled using an electrical miller (FW 100, Yusung Industrial Ltd, China) to fine powder to pass a mesh size of 0.425 mm. Finally, the dried and powdered samples were packed in paper bags and sealed in an airtight polyethylene bag and labeled before storing in a refrigerator set at 4°C for further analysis.

**Figure 1:**
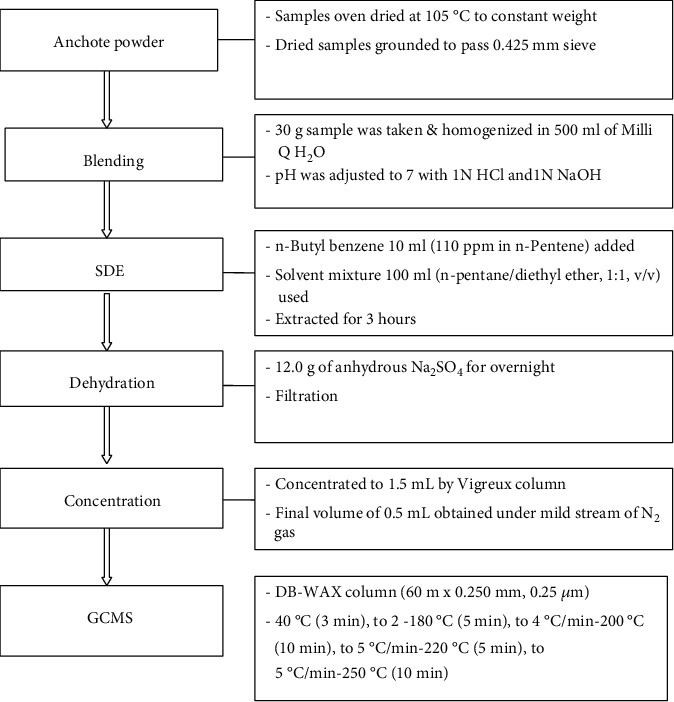
Schematic diagram for analysis of volatile flavor compounds of anchote leaf and tuber samples.

Anchote leaf and tuber samples (30 g) were homogenized in a multimixer blender (MR 350CA, Braun, Spain) and mixed with 500 ml of distilled water which were adjusted to pH 7 using 1 N NaOH and 1 N HCl. After adjusting the pH, 10 ml of n-butyl benzene (110 ppm in n-pentane) was added as an internal standard. The resultant slurry was used for extraction of volatile organic compounds with 100 ml redistilled n-pentane: diethyl ether (1 : 1, v/v) mixture, using a simultaneous distillation extraction apparatus under atmospheric pressure according to MacLeod et al. [14] for three hrs. The solvent containing the compound extract was dehydrated for 12 hrs using 10 g anhydrous Na_2_SO_4_ and then concentrated to approximately 1.5 ml using the Vigreux column (250 ml, Normschliff, Werthein, Germany). This extract was further concentrated to 0.5 ml under a gentle stream of N_2_ gas and used for gas chromatography mass spectrometry (GC-MS) analysis (Figure 1).

### 2.3. Establishment of Retention Index of N-Alkane

The standard value of retention index (RI) was determined by n-alkane mixture (C7 ~ C22). 1 *μ*L mixture of n-alkanes was analyzed to determine the retention time (RT) by GC-MS analysis following the same conditions as mentioned in Table 1. The RI of each peak was established by basic program that substituted the RT of peak of n-alkane confirmed by GC-MS chromatogram.

Retention index was used as a parameter for checking of a solute from the chromatogram by comparing the retention time of both alkanes that appeared above and below the solute.

(1) $$\begin{array}{c} {\text{RI}}_{i}=100Z+100\left\{\frac{\left(\text{Log VR }\left(i\right)-\text{Log VR }\left(Z\right)\right)}{\left(\text{Log VR }\left(Z+1\right)-\text{Log VR }\left(Z\right)\right)}\right\}, \end{array}$$

where RI*_i_* is the retention index of compound *i*, *V*_*R*(*i*)_, *V*_*R*(*Z*)_, and *V*_*R*(*Z* + 1)_ are the retention time of standard alkanes (alkanes eluted before and after the substance of interest) which bracket the substance of interest, and factor *Z* contains the number of carbon eluted, e.g., *Z* + 1 and *Z* + 2.

By definition, the retention time of an alkane is the value as a multiple of the carbon number that the compound has to be unrelated with the column solid phase, the temperature of separation, and the requirements of other chromatography. Therefore, n-alkane was indicated as standard index for CH_4_ (RI = 100), C_2_H_6_ = (RI = 200),…, C_n_H_2*n*+2_ (RI = 100_*n*_), and even anything in analysis column.

### 2.4. Chromatographic Analysis of Volatile Flavor Compounds

Chromatographic analysis was carried out using GC-MS (Model QP-2010, Shimadzu Co., Kyoto, Japan) in electron impact ionization (EI) mode. The ionization voltage was 70 eV, and the temperatures of the ion source and injector were 230°C and 250°C, respectively. The mass spectrometer was scanned from 50 to 400 m/z. The separation was done by a capillary column, DB-WAX (60 m length × 0.25 mm diameter, 0.25 *μ*m film thickness, Agilent J&W, USA). The program of oven temperature was initially started at 40°C (isothermal for 3 min), which was ramped to 180°C (isothermal for 5 min) at 2°C/min. Subsequently, it increases to 200°C (isothermal for 10 min) at 4°C/min and to 220°C (isothermal for 5 min) at 5°C/min. Finally, it reaches to 250°C (isothermal for 10 min) at 5°C/min. Helium was used as the carrier gas at a flow rate of 1 ml/min, and the sample injector volume was 1 *μ*L using 1 : 100 split ratio.

### 2.5. Identification and Quantification of Volatile Flavor Compounds

Mass spectra of volatile organic compounds were identified with the aid of our own mass spectral data and those contained within the Wiley 7, NIST 05, and FFNSC 2.0 spectral libraries of the GCMS instrument. In addition, by the comparison of retention indices to the reference data [6, 15–28]. To calculate the relative response factor (RRF), the following formula was used:

(2) $$\begin{array}{c} \text{RRF}=\left(\text{Peak Area C}/\text{Conc}.\text{C}\right)/\left(\text{Peak Area A}/\text{Conc}.\text{A}\right), \end{array}$$

where peak area C is the peak area of each component in the standard sample, Conc. C is the concentration of each component in the standard sample, peak area A is the peak area of the internal standard in the standard sample, and Conc. A is the concentration of the internal standard in the standard sample.

The quantitative analysis was carried out with the help of peak area percent of the internal standard (n-butylbenzene) using the formula [29]:

(3) $$\begin{array}{c} \text{Component content }\left(\text{mg}/\text{kg}\right)=\left(C\times 1000\text{ g}\right)/\left(A\times \text{RRF}\times B_{\text{g}}\right), \end{array}$$

where *A* is the peak area of the internal standard in the anchote sample, *B*_*g*_ is the amount of sample, and *C* is the peak area of each component in the anchote sample.

## 3. Results and Discussions

### 3.1. Volatile Compounds of Anchote Leaf

The identified volatile components, percentage of their relative peak area, and concentrations are shown in Table 2. VOCs having an area percentage above 0.5% were used for identification analysis. In anchote leaf, 30 compounds were identified, among which 16 compounds were distinguished in each of the tested accessions. These compounds are ethyl acetate, acetoin, 1,1-diethoxyethane, 3-methyl-1-butanol (E)-2-hexenal, (Z)-3-hexen-1-ol, benzaldehyde, benzyl-alcohol, phenylacetaldehyde, phenethyl-alcohol, butyrophenone, 2-methoxy-4-vinylphenol, phytone, methyl-palmitate, dibutyl-phthalate, and methyl-linolenate. Ethyl acetate 90.47% (697.13 mg/kg) was detected in a higher amount followed by phenylacetaldehyde 1.88% (14.51 mg/kg) and (E)-hex-2-enal 1.62% (12.47 mg/kg), by exhibiting >1% peak area. They accounted together (93.97% (724.11 mg/kg)). The rest (6.03%) (46.46 mg/kg) were minor quantities (<1%) of the total (770.57 mg/kg) volatile flavor fraction. The VOCs identified in anchote leaves have various applications. A monoterpene compound linalool was presented in very low concentration in accession “220563.” It is one of the major volatile components of several aromatic species used in foodstuffs as food additives and pharmacologically to cure a variety of ailments, being a sedative effect inducer, glutamatergic neurons inhibitor, anti-inflammatory, anticarcinogenic, and antiseptic [30]. Furfural has an aroma of almonds and is one of the components found in vanilla. Furfural has a wide variety of uses such as for flavoring food, as herbicide and fungicide, and affects yeast survival and biochemical enzyme activities [6]. Nonanal, 1-hexanol, (Z)-3-hexenol, linalool, and benzaldehyde were considered as important contributors to the aroma of fresh plum fruit [31]. 3-Methyl-1 butanol is a main ingredient in the production of banana oil, an ester found in nature and produced as flavoring in industry. (*Z*)-3-Hexen-1-ol is an essential aroma compound used in fruit and vegetable flavors as well as in perfumes. 1-Hexanol was used in perfume industry. Benzyl alcohol is produced naturally by many plants and commonly found in fruits and teas. It is also found in a variety of essential oils [32]. Hexanol occurs naturally after hydrolysis or enzymatic reduction reactions and was used in the flavor industry to produce fruity flavors. Benzaldehyde has a characteristic almond-like odor and is the primary component of bitter almond oil. Benzaldehyde is commonly employed to confer almond flavor to foods and scented products and sometimes used in cosmetics products [33]. Acetol or 1,1-diethoxyethane is a major flavoring component of distilled beverages, especially malt whisky. Phenylacetaldehyde is used as an ingredient in fragrances as well as in flavored cigarettes and beverages; its aroma is described as honey-like, sweet, rose, green, and grassy [34]. The aroma of ethyl acetate contributes towards the general perception of fruitiness. Dihydroactinidiolide has a sweet, tea-like odor and was used as a fragrance. Acetoin is used as a food flavoring in baked goods and as a fragrance. At very low concentrations, indole has a flowery smell and is a constituent of many flower scents such as orange blossoms and perfumes. 2-Methoxy-4-vinylphenol is an aromatic substance used as a flavoring agent and is known as one of the compounds responsible for the natural aroma of buckwheat [35]. Caryophyllene oxide, which is an oxygenated terpenoid, is also well known for its preservative characteristics in foods, drugs, and cosmetics. It has a significant central and peripheral analgesic, along with anti-inflammatory activity [36].

From the identified carbonyl compounds, phenylacetaldehyde accounted the highest amount 14.51 mg/kg (74.35%) followed by 1,1-diethoxyethane 2.78 mg/kg (14.27%), while acetoin and nonanal were quantified as 1.32 mg/kg (6.77%) and 0.90 mg/kg (4.60%), respectively. The alcohol group constituted 1.51% of the identified volatile compounds that were specified as 3-methyl-1-butanol (0.46%), [Z]-3-hexen-1-ol (0.61%), 1-hexanol (0.05%), benzyl alcohol (0.32%), 4-nonanol (0.04%), and linalool (0.04%). The remaining four functional groups such as alkanes, hydrocarbons, ketone, and terpene were detected at levels lower than 0.4%. Besides the identified functional groups, nine other volatile compounds were with no identified functional groups and categorized as miscellaneous. Among the miscellaneous (E)-2-hexenal (1.62%) which occupied the major position with >1%, the remaining compounds in content order were as follows: hexadecanoic acid <n->, phenethyl alcohol, methyl linolenate, butyrophenone, 2-methoxy-4-vinylphenol, phytone, (-)-caryophyllene oxide, and indole.

### 3.2. Volatile Compounds of Anchote Tuber

The identified VOCs in three accessions of anchote tuber that are listed according to their elution order on DB-WAX column with their number of concentrations are shown in Table 3. Fifteen volatile compounds were identified in anchote tubers from three accessions. Among the 15 identified compounds, ethyl acetate was the only major compound that accounted together for 99.15% (4498.33 mg/kg) of the total volatile flavor fraction (4536.91 mg/kg), and 0.85% (38.58 mg/kg) were reported in minor quantities (<1%).

Hexanal content is directly related to oxidative off-flavors, and the compound is easily detected because of its low odor threshold (5 ppb). Propyl acetate is commonly used in fragrances and as a flavor additive. Propyl acetate is synthesized via alcohol or acetic acid having a clear, volatile, and mobile liquid with a characteristic odor reminiscent of acetone and pears and was commonly used in fragrances and as a flavor additive. Typical odors of pyrazine compounds are responsible for the nut-like and peanut butter-like flavors, which are found in roasted barley, coffee, potato chips, and cocoa. 2,3,5-Trimethylpyrazine is categorized as cosmetic, flavor, and fragrance agents, and tetramethylpyrazine is an inhibitor of phosphodiesterase, which has been widely used for the treatment of cardiovascular diseases. Tetramethylpyrazine has also significant vascular protective properties which have been used widely for the treatment of ischemic neural disorders and cardiovascular diseases [37]. Butyrophenones are widely used drugs for the treatment of psychose and are frequently encountered in forensic chemistry and clinical toxicology. Dibutyl phthalate is an artifact extracted from plastic which often is present in extracted samples from fruit [38, 39]. It is also identified from buckwheat honey [40].

The identified volatile organic compounds in anchote leaves so far belong to the chemical classes of alcohol (7), aldehydes (3), alkanes (2), carbonyl compound (4) esters (3), hydrocarbons (1), ketones (1), terpenes (1), and miscellaneous (8) (Table 4). Esters were dominant with the highest proportion of relative peak area (91.34%) of the emitted volatile organic compounds. Ethyl acetate accounted 99.05% among the ester content (703.84 mg/kg), whereas the remaining percentage was shared by methyl palmitate (0.25%) and dibutyl phthalate (0.71%). Carbonyl compounds were the second major group, accounting 2.53% of the relative peak area. Anchote tubers so far belong to the chemical classes of alcohol (1), aldehydes (1), alkanes (1), carbonyl compounds (3), esters (2), heterocyclic compounds (1), unknown (1), and miscellaneous (5) that are present in Table 4. The order of concentration for the identified functional groups is as follows: esters > carbonyl compounds > alcohols > alkanes > aldehydes > heterocyclic compounds. Most esters have a fruity and floral flavor and may contribute to the aroma and flavor. Carbonyl compounds are widely found in food products, such as fried foods and beverages, and it is caused by the oxidation of fatty acids and higher alcohols, Strecker degradation, aldol condensation, or Maillard reactions [41]. 2-Pentanol was the only compound belonging to the alcohol group constituting 0.22% that is lower than 1% considered as a minor compound.

Aldehydes are particularly important in relation to flavor alterations and from a toxicological perspective. A particular property of the aldehyde volatile oils is their insect repellent activity due to very strong scent [6].

Alcohols, aldehydes, alkanes, carbonyl compounds, and esters were found in both the leaf and tuber samples. Comparative profile of VOCs showed the group with the highest percentage of compounds which was detected in the ester group, accounting 91.34% for leaves and 99.16% for tubers, followed by carbonyl compounds and alcohols in both leaf and tuber samples.

## 4. Conclusion

The study of volatile organic compounds in the leaf and tuber parts of the three anchote accessions showed that there are several bioactive volatile components present, which could be isolated and used for various purposes. In general, this study confirms the potential of anchote for various biochemical applications in foods and in folk medicine. Therefore, further studies on the extraction and structure elucidation of various important VOCs from different parts of anchote are essential to promote effective utilization of underexploited genetic resources for more specific and valuable applications in the field of human health and food industries. Since the VOC extraction of anchote tuber and leaf was done for only three accessions, it was difficult to identify several new VOCs using SDE extraction and GC-MS analysis.

## Figures and Tables

**Table 1:** Retention time of n-alkane mixture for GC-MS retention index.

Alkanes	Retention time	Alkanes	Retention time
C_7_	8.405	C_15_	62.875
C_8_	13.095	C_16_	68.877
C_9_	19.775	C_17_	74.637
C_10_	27.423	C_18_	81.002
C_11_	35.188	C_19_	86.341
C_12_	42.689	C_20_	92.825
C_13_	49.808	C_21_	98.567
C_14_	56.528	C_22_	104.059

**Table 2:** Volatile organic compounds identified in anchote leaf.

No.	Compound name	MF^a^	223090			220563			230566		
			Area%	mg/kg	RI^b^	Area%	mg/kg	RI^b^	Area%	mg/kg	RI^b^
1	Ethyl acetate	C_4_H_8_O_2_	4.52	19.58	606	66.58	335.49	824	4.13	18.17	606
	Ethyl acetate	C_4_H_8_O_2_	0.10	0.45	899	—	—	—	73.50	322.97	820
	Ethyl acetate	C_4_H_8_O_2_	—	—	—	—	—	—	0.11	0.47	898
2	Acetoin	C_4_H_8_O_2_	0.10	0.44	710	0.11	0.53	710	0.08	0.35	710
3	1,1-Diethoxyethane	C_6_H_14_O_2_	0.19	0.81	727.22	0.17	0.87	727	0.25	1.10	727
4	3-Methyl-1-butanol	C_5_H_12_O	0.28	1.19	736	0.19	0.98	736	0.31	1.36	736
5	Hexanal	C_6_H_12_O	—	—	—	—	—	—	0.07	0.32	801
6	Furfural	C_5_H_4_O_2_	0.18	0.79	837	0.15	0.78	837	—	—	—
7	(E)-2-Hexenal	C_6_H_10_O	0.98	4.25	856	0.79	3.96	857	0.97	4.26	856
8	(Z)-3-Hexen-1-ol	C_6_H_12_O	0.46	1.98	858	0.27	1.38	859	0.31	1.35	858
9	Ethyl benzene (EB)	C_8_H_10_	—	—	—	0.05	0.24	863	—	—	—
10	1-Hexanol	C_6_H_14_O	0.08	0.35	872	—	—	—	—	—	—
11	Benzaldehyde	C_7_H_6_O	0.11	0.47	965	0.10	0.53	965	0.11	0.50	965
12	Benzyl alcohol	C_7_H_8_O	0.10	0.41	1036	0.13	0.68	1036	0.31	1.37	1036
13	Phenylacetaldehyde	C_8_H_8_O	0.60	2.60	1047	1.49	7.49	1047	0.77	3.40	1047
	Phenylacetaldehyde	C_8_H_8_O	—	—	—	0.20	1.01	1051	—	—	—
IS^c^	Butylbenzene	C_10_H_14_	7.70	33.33	1059	6.61	33.33	1059	7.57	33.33	1059
14	4-Nonanol	C_9_H_20_O	—	—	—	0.06	0.29	1093	—	—	—
15	Linalool	C_10_H_18_O	—	—	—	0.05	0.27	1099	—	—	—
16	Nonanal	C_9_H_18_O	0.08	0.35	1105	—	—	—	0.12	0.55	1105
17	Phenethyl alcohol	C_8_H_10_O	0.11	0.49	1113	0.34	1.71	1113	0.35	1.54	1113
18	Butyrophenone	C_10_H_12_O	0.09	0.40	1255	0.16	0.79	1255	0.09	0.38	1255
19	Indole	C_8_H_7_N	—	—	—	—	—	—	0.05	0.23	1292
20	2-Methoxy-4-vinylphenol	C_9_H_10_O_2_	0.07	0.30	1310	0.14	0.70	1310	0.11	0.47	1310
21	Beta-ionone	C_13_H_20_O	—	—	—	0.09	0.46	1481	0.06	0.27	1481
22	Dihydroactinidiolide	C_11_H_16_O_2_	—	—	—	0.05	0.25	1533	0.10	0.44	1533
23	(-)-Caryophyllene oxide	C_15_H_24_O	—	—	—	0.12	0.60	1588	—	—	—
24	Phytone	C_18_H_36_O	0.05	0.23	1841	0.12	0.59	1840	0.09	0.39	1799
25	Methyl palmitate	C_17_H_34_O_2_	0.10	0.43	1922	0.18	0.93	1922	0.09	0.38	1900
26	Dibutyl phthalate	C_16_H_22_O_4_	0.28	1.21	1950	0.41	2.08	1950	0.38	1.69	1899
27	Hexadecanoic acid <n->	C_16_H_32_O_2_	—	—	—	0.40	2.02	1955	0.79	3.46	1899
28	Eicosane <n->	C_20_H_42_	—	—	—	—	—	—	0.05	0.20	2000
29	Methyl linolenate	C_19_H_32_O_2_	0.19	0.81	2097	0.23	1.18	2096	0.29	1.28	1999
30	Docosane <n->	C_22_H_46_	—	—	—	0.06	0.31	2200	—	—	—

^a^Molecular formula, ^b^retention index, and ^c^internal standard.

**Table 3:** Volatile flavor compounds identified in anchote tuber.

No.	Compound name	MF^a^	223090			220563			230566		
			Area%	mg/kg	RI^b^	Area%	mg/kg	RI^b^	Area%	mg/kg	RI^b^
1	2-Pentanol	C_5_H_12_O	0.20	8.09	701	0.19	1.98	701	—	—	—
2	Heptane	C_7_ H_16_	—	—	—	—	—	—	0.14	0.76	699
3	Acetoin	C_4_H_8_O_2_	0.12	4.62	710	0.12	1.26	710	0.11	0.61	709
4	Pyrrole	C_4_H_5_N	—	—	—	—	—	—	0.05	0.26	750
5	1,1-Diethoxyethane	C_6_H_14_O_2_	0.13	5.15	727	0.25	2.53	727	0.34	1.86	727
	1,1-Diethoxyethane	C_6_H_14_O_2_	0.06	2.23	737	0.05	0.54	737	—	—	—
	1,1-Diethoxyethane	C_6_H_14_O_2_	0.06	2.35	916	—	—	—	—	—	—
6	Hexanal	C_6_H_12_O	—	—	—	—	—	—	0.12	0.67	801
7	Ethyl acetate	C_4_H_8_O_2_	81.77	3224.81	822	80.47	823.19	822	79.16	433.32	822
	Ethyl acetate	C_4_H_8_O_2_	0.08	3.10	976	0.22	2.30	899	0.14	0.79	899
	Ethyl acetate	C_4_H_8_O_2_	0.27	10.81	994	—	—	—	—	—	—
8	Unknown	C_9_H_20_O_4_	—	—	—	0.05	0.48	904	—	—	—
9	2-Pentyl furan	C_9_H_14_O	—	—	—	—	—	—	0.10	0.56	991
10	Benzaldehyde	C_7_H_6_O	—	—	—	—	—	—	0.10	0.54	965
11	n-Propyl acetate	C_5_H_10_O_2_	—	—	—	0.07	0.68	976	—	—	—
12	2,3,5-Trimethyl pyrazine	C_7_H_10_N_2_	—	—	—	—	—	—	0.06	0.30	1001
13	Tetramethyl pyrazine	C_8_H_12_N_2_	—	—	—	—	—	—	0.38	2.11	1084
IS^c^	Butylbenzene	C_10_H_14_	0.84	33.33	1059	3.25	33.33	1059	6.08	33.33	1059
14	Butyrophenone	C_10_H_12_ O	—	—	—	—	—	—	0.08	0.45	1255
15	Dibutyl phthalate	C_16_H_22_O_4_	—	—	—	0.05	0.55	1950	—	—	—

^a^Molecular formula, ^b^retention index, and ^c^internal standard.

**Table 4:** Content of functional groups in identified volatile components from anchote leaves and tubers.

Functional groups	No. of compounds		mg/kg		Relative peak area%	
	Leaf	Tuber	Leaf	Tuber	Leaf	Tuber
Alcohol	6	1	1.51	0.22	11.62	10.07
Aldehyde	3	1	0.44	0.01	3.39	0.67
Alkane	2	1	0.07	0.02	0.51	0.76
Carbonyl compound	4	3	2.53	0.48	19.51	21.69
Ester	3	2	91.34	99.16	703.84	4499.01
Heterocyclic compounds	—	1	—	0.01	—	0.26
Hydrocarbon	1	—	0.03	—	0.24	—
Ketone	1	—	0.09	—	0.73	—
Terpene	1	—	0.09	—	0.69	—
Miscellaneous	9	5	3.90	0.09	30.04	3.97
Unknown	—	1	—	0.01	—	0.48
Total	30	15	100	100	770.57	4536.92

## Data Availability

The data that support the findings of this study are available upon request to the corresponding author.
